# Ghrelin Is a Regulator of Glucagon-Like Peptide 1 Secretion and Transcription in Mice

**DOI:** 10.3389/fendo.2017.00135

**Published:** 2017-06-19

**Authors:** Andreas Lindqvist, Liliya Shcherbina, Ann-Helen Thorén Fischer, Nils Wierup

**Affiliations:** ^1^Department of Clinical Sciences, Lund University Diabetes Centre, Malmö, Sweden

**Keywords:** ghrelin, glucagon-like peptide 1, GLUTag cells, oral glucose tolerance test, GHS-R1a

## Abstract

The gut hormones ghrelin, glucagon-like peptide 1 (GLP-1), and glucose-dependent insulinotropic peptide (GIP) have been intensively studied for their role in metabolism. It is, however, not well known whether the hormones interplay and regulate the secretion of each other. In this study, we studied the effect of ghrelin on GLP-1, GIP, and insulin secretion during an oral glucose tolerance test (OGTT) in mice. Intravenous administration of ghrelin caused increased GLP-1 secretion during the OGTT. On the other hand, ghrelin had no effect on circulating levels of glucose, insulin, and GIP. Furthermore, ghrelin treatment reduced proglucagon mRNA expression in GLUTag cells. The effect of ghrelin on GLP-1 secretion and proglucagon transcription was reinforced by the presence of GHS-R1a in human and mouse ileal L-cells, as well as in GLUTag cells. In summary, ghrelin is a regulator of GLP-1 secretion and transcription, and interfering with GHS-R1a signaling may be a way forward to enhance endogenous GLP-1 secretion in subjects with type 2 diabetes.

## Introduction

The gastrointestinal tract harbors an array of hormones and regulatory peptides, involved in control of processes ranging from food intake to glucose homeostasis (1). Although the biological significance of the major gut hormones is well established, the interplay between different gut hormones is not well known and needs further investigation. Glucagon-like peptide 1 (GLP-1) is a product of the proglucagon gene produced by L-cells in the distal intestine, mainly in the ileum and colon. GLP-1 is known to increase insulin secretion (2) and beta cell proliferation (3) and to inhibit intestinal motility (4), gastric emptying (5), and food intake (6). GLP-1 chemistry is an area targeted for the treatment of type 2 diabetes (T2D) and GLP-1 analogs and inhibitors of the GLP-1-degrading enzyme DPP-IV are successfully used in clinical practice (7). Glucose-dependent insulinotropic peptide (GIP) is, on the other hand, produced in K-cells mainly located to the proximal parts of the small intestine. GIP has many effects that are similar to those of GLP-1 including stimulating insulin secretion (8, 9) and promoting beta cell proliferation (10, 11). Increased understanding of the mechanisms regulating incretin secretion is important to understand normal physiology and may pave the way for new targets for the treatment of T2D. Ghrelin is a 28-amino acid peptide hormone produced by P/D1 cells in the oxyntic mucosa of the stomach (12), in the upper small intestine (13), and in a distinct cell type in the pancreatic islets (14). Normoglycemic lean humans have elevated ghrelin levels in response to fasting and the levels decrease upon meal ingestion (15). Central and peripheral administration of ghrelin in rodents and humans has been shown to increase food intake (16–18) and to inhibit insulin secretion (19). Furthermore, ghrelin has been shown to inhibit insulin secretion in isolated mouse islets (20, 21) and in clonal beta cells (22, 23). Obese individuals have lower fasting levels of ghrelin (24) and deranged suppression of ghrelin levels upon meal ingestion (25). Furthermore, lean T2D patients have higher levels of fasting ghrelin, whereas obese T2D subjects have lower levels of fasting ghrelin, than do normoglycemic lean control subjects (26). The GLP-1 analog, exendin-4 reduces ghrelin levels in fasting rats (27) and GLP-2, another product of the proglucagon gene, suppresses ghrelin secretion in humans (28). Furthermore, ghrelin was reported to attenuate the effects of GLP-1 on food intake (29). Two studies have shown that ghrelin regulates GLP-1 secretion and production, but the available data are somewhat contradictory (30, 31). Here, we aimed to assess the effect of intravenously administered ghrelin on GLP-1 and GIP secretion in response to an oral glucose tolerance test (OGTT). Our results suggest that intravenous administration of ghrelin increases GLP-1 secretion during an OGTT and that ghrelin affects proglucagon transcription *in vitro* in GLUTag cells.

## Materials and Methods

### Mice

Female C57BL/6NTac mice (approximately 25 g; 4–5 weeks of age) were housed in climate-controlled rooms (23 ± 1°C) with a 12:12 h light–dark cycle. Food and water were provided *ad libitum* unless otherwise stated. All the experiments in this study were approved by and performed in accordance with the Animal Ethics Committee, Lund and Malmö, Sweden (Ethical permit number M458-12).

### Oral Glucose Tolerance Test

The mice were fasted for 4 h before the oral glucose tolerance test (OGTT). OGTTs were performed at 1300. The mice were anesthetized using an intraperitoneal injection of Hypnorm/Dormicum (10 µl/g BW; fentanyl 0.315 mg/ml, fluanison 10 mg/ml, and midazolam 5 mg/ml). Basal blood samples (40 µl) were collected through retro-orbital puncture and ghrelin [50 nmol/kg (21); Phoenix Pharmaceuticals, Burlingame, CA, USA] or saline was injected intravenously in a tail vein 5 min prior to glucose administration (32, 33) (*n* = 18 and *n* = 15, respectively). At time 0, glucose [3 mg/g body weight (32, 33)] was given orally through gavage. Blood samples (40 μl/time point) were collected at 10, 20, 30, 60, and 90 min through retro-orbital puncture. After glucose tolerance tests, the mice were kept overnight under a heating lamp for recovery.

### Blood Collection

Blood was collected in chilled tubes supplemented with 500 KIU/ml Aprotinin (Trasylol^®^; Leverkusen, Germany) and 100 µmol/ml of the DPP-IV inhibitor Diprotin A (Sigma Aldrich, St. Louis, MO, USA). Plasma (1,500 × *g*, 3 min, 4°C) was stored at −80°C until analysis.

### Hormone Analyses

Active GLP-1, total GIP, and total ghrelin were analyzed using ELISAs from Millipore (Darmstadt, Germany). Insulin was analyzed using an ELISA from Mercodia (Uppsala, Sweden), and glucose was analyzed using a commercially available kit (Infinity Glucose Oxidase) from Thermo Fisher Scientific (Lexington, MA, USA). Assays were performed according to the instructions provided by the manufacturers.

### Cell Culture

The GLUTag cell line (provided by Dr. Daniel J. Drucker, Mount Sinai Hospital, Toronto, ON, Canada) was originally isolated from a glucagon-producing enteroendocrine tumor in mice. GLUTag cells were routinely cultured in Dulbecco’s modified Eagle’s medium, 1 g/l glucose, supplemented with 10% FBS, and 2 mM glutamine. For ghrelin treatment, cells were seeded in 24-well plates at a density of 250,000 cells/well and cultured for 24 h. Thereafter, medium was replaced by new medium with or without ghrelin at concentrations of 10 nM, 100 nM, and 1 µM and cells were incubated for 24 h.

### Quantitative Real-time PCR

RNA was extracted from GLUTag cells using a commercially available kit (Nucleo Spin RNA II, Macherey Nagel, Bethlehem, PA, USA). cDNA was generated using a RevertAid First Strand cDNA Synthesis kit (Thermo Fisher Scientific, Waltham, MA, USA). Real-time PCR was run using TaqMan^®^ assays [GLP-1, Mm1269055_m1; peptidylpropyl isomerase A (PPIA) Mm02342429_g1]. 25 ng of cDNA was run under the following conditions: 1 cycle of 50°C for 2 min and 95°C for 10 min followed by 40 cycles of 95°C for 15 s and 60°C for 1 min. The mRNA expression was calculated using the 2^−ΔΔ(Ct)^ formula and expressed as arbitrary units in relation to PPIA expression that was used as reference gene.

### Tissue Collection

Mouse ileum was collected from female C57BL/6NTac mice (approximately 25 g body weight), and specimens from human terminal ileum were taken during colonoscopy as previously detailed (13). The studies were approved by the Human Ethics Committee in Lund.

### Immunohistochemsitry

Specimens of human and mouse ileum were fixed in 4% paraformaldehyde and embedded in paraffin. Sections (6 µm) were cut on a microtome, and slides were incubated with previously characterized primary antibodies for ghrelin receptor (GHS-R1a) (rabbit antibody; code: H-001-62, dilution: 1:400, Phoenix Pharmaceuticals, Burlingame, CA, USA), proglucagon (guinea pig antibody; code: M7807, dilution: 1:5,000, EuroDiagnostika, Malmö, Sweden), and GIP (goat antibody; code: sc-23554; dilution 1:500, Santa Cruz Biotechnology, Houston, TX, USA) overnight at 4°C. Thereafter, slides were incubated with secondary antibodies [donkey anti-rabbit Cy2 (1:400) for GHS-R1a, donkey anti-guinea pig Texas Red (1:400) for GLP-1, and donkey anti-goat Texas Red (1:400) for GIP] for 1 h at room temperature. GLUTag cells were cultured on cover slips and incubated with GHS-R1a antibody (code: 00020; dilution: 1:400) overnight at 4°C. Secondary antibody (donkey anti-rabbit Cy2; 1:400) was applied and incubated for 1 h at room temperature. Nuclei was stained with 1 µM DAPI (Thermo Fisher Scientific, Waltham, MA, USA).

### Statistics

Data are presented as mean ± SEM. Statistical significance was calculated using one-way or two-way ANOVA where appropriate using the software GraphPad Prism 6 (GraphPad Software Inc., La Jolla, CA, USA). Differences were considered significance if *p* < 0.05.

## Results

### Effect of Intravenous Ghrelin on GLP-1, GIP, and Insulin Release during an OGTT

**Figure 1:**
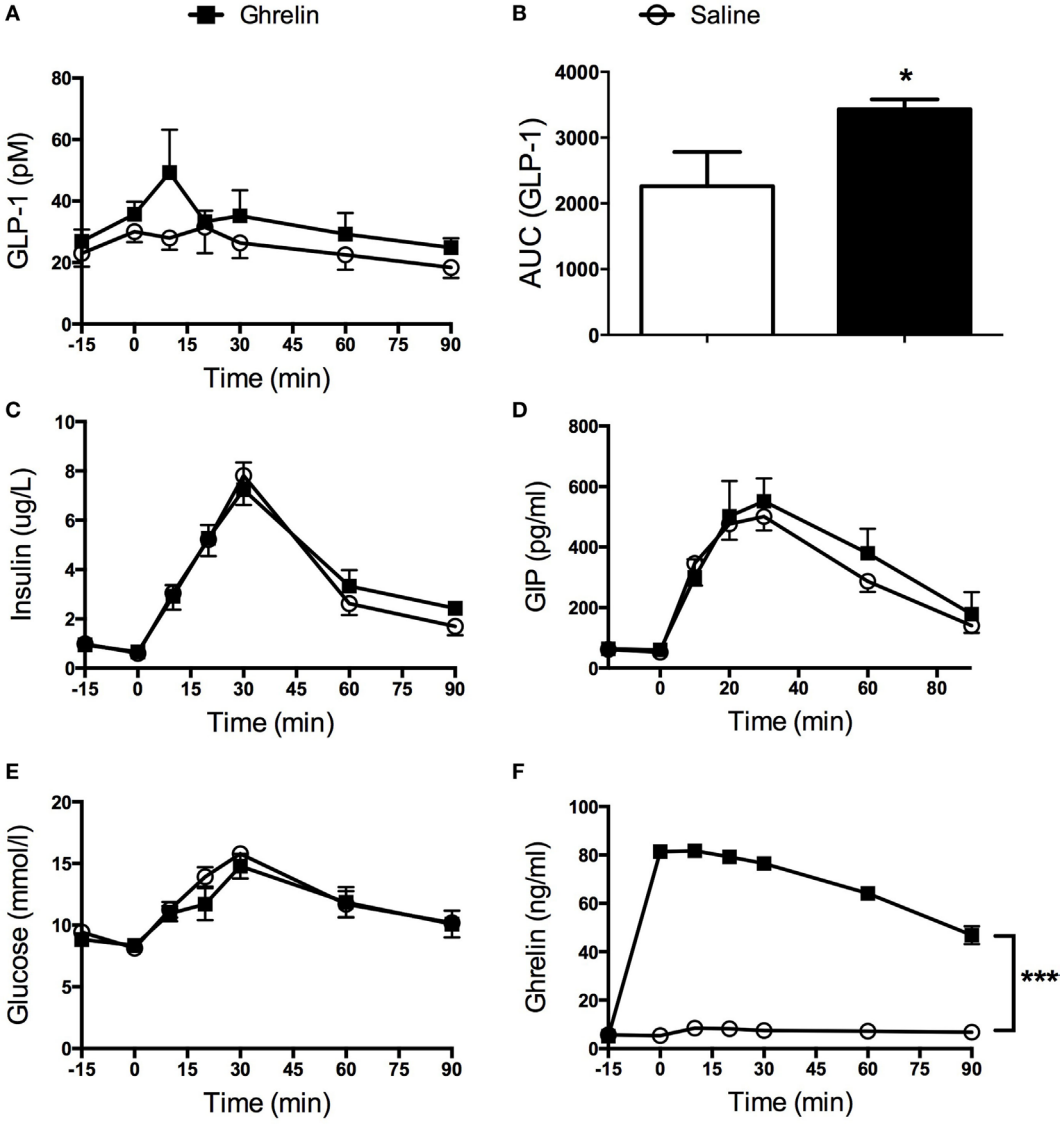
Glucagon-like peptide 1 (GLP-1) secretion was increased in mice receiving ghrelin intravenously (50 nmol/kg) **(A)**. AUC calculations revealed a statistically significant increase in GLP-1 after ghrelin injection **(B)**. Insulin **(C)**, glucose-dependent insulinotropic peptide (GIP) **(D)**, and glucose **(E)** were unaffected by ghrelin treatment. Hyperghrelinemia in the mice was confirmed by significantly elevated levels of circulating ghrelin in the group of mice having received intravenous administration of ghrelin **(F)**. *p < 0.05; ***p < 0.001. All statistical analyses were performed using two-way ANOVA except for **(B)** where Student’s *t*-test was applied.

Ghrelin had no effect on basal GLP-1 levels, but glucose-stimulated GLP-1 secretion was found to be transiently increased in mice that had received intravenous administration of ghrelin (Figure 1A). Thus, AUC for GLP-1 was increased approximately 1.5-fold (*p* < 0.05; Figure 1B). On the other hand, basal and glucose-stimulated plasma levels of insulin, GIP and glucose (Figures 1C–E) were unaffected by ghrelin administration. Elevated circulating ghrelin levels in ghrelin-treated mice were confirmed (Figure 1F).

### Effects of Ghrelin on Proglucagon mRNA in GLUTag Cells

**Figure 2:**
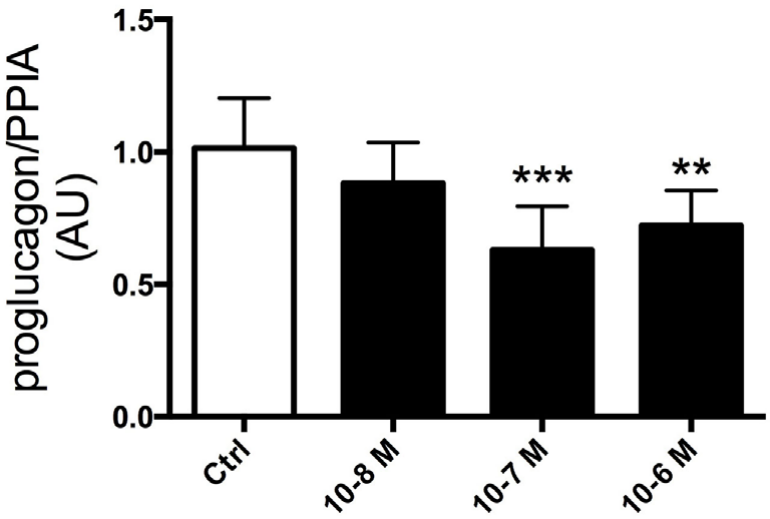
GLUTag cells were incubated with 10^−6^, 10^−7^, or 10^−8^ M ghrelin for 24 h. Cells treated with 10^−6^ and 10^−7^ M ghrelin displayed reduced proglucagon mRNA expression compared to control cells. **p < 0.01; ***p < 0.001. Statistical analysis was performed using one-way ANOVA. Experiments were repeated in eight passages of cells (*n* = 8).

Having established that ghrelin affects GLP-1 plasma levels, we next assessed whether ghrelin impacts on proglucagon transcription. To this end, GLP-1-expressing GLUTag cells were used as a model. Ghrelin was added to the media at different concentrations (10^−6^, 10^−7^, and 10^−8^ M) and cells were cultured for 24 h. This revealed that addition of ghrelin (10^−6^ or 10^−7^ M) resulted in reduced proglucagon mRNA expression compared with vehicle-treated control cells (*p* < 0.01 and *p* < 0.005, respectively; Figure 2).

### GHS-R1a Expression in Mouse and Human Ileum

**Figure 3:**
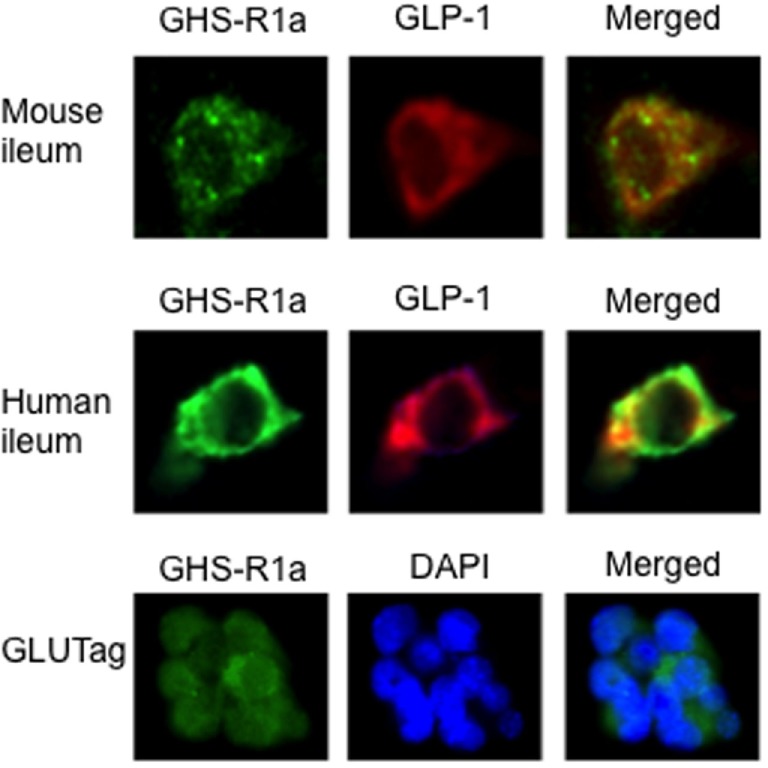
The presence of the ghrelin receptor, GHS-R1a, was confirmed in mouse and human ileum using immunohistochemistry. Double staining with glucagon-like peptide 1 (GLP-1) revealed that GHS-R1a is colocalized with GLP-1 in mouse and human ileum (upper panel). GHS-R1a immunoreactivity was also evident in GLUTag cells (lower panel).

Our data on the effect of ghrelin on GLP-1 secretion imply that L-cells express ghrelin receptors (GHS-R1a). To test this, we double stained mouse and human ileal sections for GHS-R1a and proglucagon. This revealed that GLP-1-producing L-cells harbored GHS-R1a expression in both species (Figure 3). On the other hand, GIP-producing K-cells were devoid of GHS-R1a (data not shown). Furthermore, stainings for GHS-R1a confirmed GHS-R1a expression also in GLUTag cells (Figure 3).

## Discussion

The gut hormones ghrelin and GLP-1 have been extensively studied the last decade. However, it is still not fully understood how they interplay. Here, we show that intravenously administered ghrelin stimulates secretion of GLP-1 *in vivo* in mice, as well as that ghrelin affects GLP-1 transcription in an *in vitro* model of L-cells. On the other hand, ghrelin had no effect on plasma levels of GIP, insulin, or glucose.

Our data on the stimulatory effect of ghrelin on GLP-1 gain support from a recent study by Gagnon et al. who reported stimulatory effects of ghrelin on GLP-1 secretion *in vivo* in mice and *in vitro* in GLUTag and NCI-H716 cell lines *via* MAPK-dependent pathways (30). Furthermore, the authors demonstrated a role for endogenous ghrelin as a regulator of GLP-1 since treatment with a ghrelin receptor antagonist reduced circulating GLP-1 levels. In contrast to our present data, Gagnon et al. found ghrelin administration to reduce glucose levels paralleled by a minute increase in insulin secretion. Interestingly, these effects were absent in mice lacking the GLP-1 receptor and in wild-type mice cotreated with a GLP-1 receptor antagonist (30). The divergent data with respect to insulin and glucose levels may be related to differences in study design between the study by Gagnon et al. and the present study. First, different doses of ghrelin were used; Gagnon et al. used a fourfold higher dose of ghrelin (200 vs 50 nmol/kg). Second, the timing of ghrelin injection differed. Gagnon et al. injected ghrelin at −15 min, whereas in the present study, ghrelin was injected at −5 min. Third, the administration route differed. Whereas ghrelin was administered intravenously in the present study, Gagnon et al. administered ghrelin intraperitoneally. Also, in the present study, active GLP-1 was measured, while Gagnon et al. measured total GLP-1. Although it is difficult to compare the effect of acute administration of ghrelin with that of life long absence of the ghrelin receptor, our data and the data by Gagnon et al. are not in accordance with the data, presented by Xu et al., on increased circulating levels of GLP-1 in GHS-R1A null mice. However, it needs to be mentioned that Xu et al. only reported basal levels of GLP-1 and did not assess GLP-1 levels during a glucose tolerance test (31).

The two previous reports also assessed GLP-1 secretion from cell lines, but there is no consensus on the direction of the effect. Thus, Xu et al. (31) showed that ghrelin suppresses active GLP-1 secretion in STC-1 cells, whereas Gagnon et al. found ghrelin to increase GLP-1 secretion in GLUTag cells and NCI-H716 cells (30).

Our present *in vivo* data are in agreement with a study in humans by Tong et al. (34) who elegantly showed that during a meal tolerance test, infusion of a high dose of ghrelin increased the postprandial response of GLP-1, without affecting GIP, peptide YY, or glucagon. On the other hand, ghrelin had no effect on GLP-1 levels during an intravenous glucose tolerance test (IvGTT) (34). Furthermore, insulin secretion rate adjusted for glucose was reduced by ghrelin infusion.

In this study, ghrelin administration had no effect on insulin and glucose levels during the OGTT. This is in contrast to previous reports showing that ghrelin inhibits insulin secretion in different models and settings, including IvGTT in mice (21), *in vitro* studies in isolated islets and clonal cells (21–23, 35), as well as *in vivo* in humans (34, 36). To the best of our knowledge, the effect of intravenously administered ghrelin on insulin secretion during an OGTT in mice has not been reported previously. Fusco et al. administered ghrelin intravenously to obese patients with or without polycystic ovary syndrome and found ghrelin to suppress insulin secretion during an OGTT (37). However, incretin levels were not measured (37). We suggest that a possible explanation for the lack of effect of ghrelin on insulin in this study may be a result of the stimulatory actions of GLP-1 masking the inhibitory effect of ghrelin on the beta cell.

Furthermore, intravenous ghrelin administration had no effect on circulating levels of GIP during the OGTT. This is in line with the data presented by Tong et al. in healthy human subjects (34). As the half-life of ghrelin is approximately 85 min (38), it seems unlikely that this would contribute to the observations presented here.

We also showed that ghrelin reduced proglucagon mRNA expression in GLUTag cells, an *in vitro* model of L-cells. This observation was unexpected in light of the stimulatory effects of ghrelin on GLP-1 secretion *in vivo*. There is no ready explanation for the divergent data, and it is not known whether ghrelin has the same effect in L-cells *in vivo*. However, our present data gain support from similar observations in STC-1 cells (31). It should be mentioned that the effect on circulating levels of GLP-1 *in vivo* was evident within minutes, whereas the effect of ghrelin on proglucagon mRNA in GLUTag cells was seen after 24 h of culture. Furthermore, the effect of ghrelin on proglucagon mRNA integrity is not known. Further studies are needed to understand the impact of ghrelin on GLP-1 transcription and production.

Nevertheless, our functional data gain support from our confirmatory finding of GHS-R1a expression in L-cells in human and murine ileum, as well as in GLUTag cells. Thus, providing anatomical prerequisites for a direct effect of ghrelin on L-cells. Gagnon et al. demonstrated GHS-R1a mRNA expression in GLUTag and NCI-H716 cells (30). In this study, we confirm the previous findings of Xu et al. who demonstrated the presence of GHS-R1a in L-cells in murine ileum (31). The lack of effect of ghrelin on GIP secretion shown here and reported by Tong et al. (34) is most likely explained by our present finding of lack of GHS-R1a in K-cells.

In summary, during an oral glucose challenge, intravenously administered ghrelin stimulates circulating levels of GLP-1, without affecting levels of GIP, insulin, or glucose. Furthermore, ghrelin affects GLP-1 transcription and we confirm previous observations that murine L-cells express GHS-R1a and also show that human ileal L-cells express GHS-R1a.

## Ethics Statement

The experiments were approved by the Animal Ethics Committee, Lund and Malmö, Sweden.

## Author Contributions

AL performed analyses and co-wrote the manuscript. LS performed analyses. A-HF performed surgeries. NW conceptualized the study and wrote the manuscript.

## Conflict of Interest Statement

The authors declare that the research was conducted in the absence of any commercial or financial relationships that could be construed as a potential conflict of interest.
